# Total occlusion of left main coronary artery. Treatment and surgical aspects

**DOI:** 10.1186/1749-8090-8-S1-P111

**Published:** 2013-09-11

**Authors:** SS Aboul-Hassan, I Szwedo, A Szymańska, P Kwinecki, R Cichoń

**Affiliations:** 1 Heart Diseases Center MEDINET, Wroclaw, Poland

## Background

This study is to evaluate the early results treatment of patients with total occlusion of the left main coronary artery. Total occlusion of the left main coronary artery is a very rare finding in patients with acute coronary syndrome. In these cases, patients present various clinical symptoms, however the symptoms and the survival of these patients depend on the development of collaterals and adequate medical intervention.

## Methods

Between January 2002 till May 2013, four patients with acute coronary syndrome caused by total occlusion of the left main coronary artery underwent emergent coronary artery bypass grafting(CABG). All patients presented chest pain, signs of cardiogenic shock with CK-MB level above100 ng/ml on admission. IABP was inserted and emergent PCI and consecutive surgery within 24 hrs were performed. One patient out of the four underwent on-pump beating heart CABG, the rest of the patients underwent classical CABG with induced cardiac arrest using blood cardioplegia with maintained normothermia. Early postoperativeevaluation was performed in terms of: ejection fraction, bleedings, CK-MB level and deaths.

## Results

Three patients, who underwent classical CABG with induced cardiac arrest using blood cardioplegia with maintained normothermia, survived, whereas patient who underwent on-pump beating heart CABG died in the post-operative period due to hemodynamic failure with an ineffective response to reanimation.

## Conclusion

It is very difficult to treat such patients with acute coronary syndrome complicated with cardiogenic shock caused by total occlusion of left main coronary artery. Such patients could develop massive myocardial infarction and should be treated immediately with emergent PCI, IABP and emergent CABG within 24 hours.On-pump beating heart CABG is possible in these patients, however we recommend treating such highly risky patients by CABG with induced cardiac arrest using blood cardioplegia with maintained normothermia.

